# Fecal microbiota transplantation to reduce immune activation in ART-treated people with HIV with low CD4/CD8 ratio: protocol for the single-blind, randomized, placebo-controlled Gutsy study (CIHR/CTN PT038)

**DOI:** 10.1186/s13063-025-09345-0

**Published:** 2025-12-13

**Authors:** Stéphane Isnard, Carolina A. Berini, Seema Nair Parvathy, Hansen Feng, Orthy Aiyana, Léna Royston, Tsoarello Mabanga, Peter L. Lakatos, Talat Bessissow, Marina B. Klein, Bertrand Lebouché, Cecilia T. Costiniuk, Bertrand Routy, Michael S. Silverman, Jean-Pierre Routy

**Affiliations:** 1 Infectious Diseases and Immunity in Global Health Program, Research Institute, McGill University Health Centre, Montréal, QC Canada; 2 Department of Medicine, Division of Infectious Diseases and Chronic Viral Illness Service, McGill University Health Centre, Montréal, QC Canada; 3 CIHR Canadian HIV Trials Network, Vancouver, BC Canada; 4 Department of Medicine, Division of Infectious Diseases, Western University, London, Ontario Canada; 5 Lawson Research Institute, St Joseph’s Health Care, London, Ontario Canada; 6 Division of Infectious Diseases, Geneva University Hospitals, Geneva, Switzerland; 7 Division of Gastroenterology, McGill University Health Center, Montréal, QC Canada; 8 Centre for Outcomes Research and Evaluation, Research Institute of the McGill University Health Centre, Montréal, QC Canada; 9 Department of Family Medicine, McGill University Health Centre, Montréal, QC Canada; 10 Centre de Recherche du Centre Hospitalier de L’Université de Montréal, Montreal, QC Canada; 11 Hemato-oncology division, Centre Hospitalier de l’Université de Montréal (CHUM), Montreal, QC, Canada; 12 Division of Infectious Diseases, St Joseph’s Health Care, London, ON, Canada; 13 Division of Hematology, McGill University Health Centre, Montréal, QC Canada

**Keywords:** HIV, Gut microbiota, Fecal microbiota transplantation, Antiretroviral therapy, Immune activation, Inflammation, Microbial translocation, Randomized control trial, Gut mucosa

## Abstract

**Background:**

Despite antiretroviral therapy (ART) controlling HIV viral replication, people with HIV (PWH) remain at risk for inflammatory non-AIDS comorbidities. Factors contributing to comorbidities in PWH on ART include spontaneous release of HIV products, CMV co-infection, microbial translocation, and gut dysbiosis, each driving systemic T-cell activation. In addition to ART, novel gut microbiota-modulating therapies could reduce epithelial gut permeability, microbial translocation, and immune activation. Fecal microbiota transplantation (FMT) from healthy volunteer is a promising therapy to counteract dysbiosis, protect from gut barrier damage, and lower systemic immune activation.

**Methods:**

The Gutsy study is a single-blind, randomized, placebo-controlled clinical trial evaluating the effects of FMT in PWH on ART for more than 3 years, with a viral load below 50 copies/mL, a CD4 count above 200 cells/mL, and a CD4/CD8 ratio below 1.0. All participants undergo a bowel cleanse before receiving FMT or placebo capsules. In the treatment group, 10 participants receive a bowel cleanse then two high doses of FMT delivered via 30 to 40 capsules twice, 3 weeks apart. The placebo group of 10 participants receive a bowel cleanse and capsules filled with microcrystalline cellulose for equivalence in weight and color, administered under the same time course. Peripheral blood mononuclear cells (PBMCs) and stool samples are collected at each visit: before bowel cleanse (baseline 1), before the first (baseline 2) and the 2nd (visit 4) FMT/placebo, 6 weeks (visit 5) and 12 weeks (visit 6) after the first FMT/placebo; colon biopsies are obtained at visits 3 and 6 in an optional sub-study. The primary objective is to assess the effect of FMT on plasma markers of gut epithelial permeability. Secondary objectives include microbial translocation, immune activation, and HIV latent reservoir biomarkers.

**Discussion:**

We hypothesize that large-dose FMT in capsules, but not placebo capsules, will increase the abundance of beneficial microbes in the gut of PWH on ART, leading to decreased gut damage markers and reduced immune activation. The results of the Gutsy pilot study will inform for the calculation of sample size of larger definitive randomized clinical trials assessing the influence of FMT on immune activation in PWH.

**Trial registration:**

ClinicalTrials.gov NCT06022406. Registered on 2024-08-01. https://clinicaltrials.gov/study/NCT06022406?cond=HIV&term=Gutsy&rank=1.

**Supplementary Information:**

The online version contains supplementary material available at 10.1186/s13063-025-09345-0.

## Administrative information

**Table:** 

Title {1}	Fecal microbiota transplantation to reduce immune activation in ART-treated people with HIV with low CD4/CD8 ratio: protocol for the single-blind, randomized, placebo-controlled Gutsy study (CIHR/CTN PT038)
Trial registration {2a and 2b}	ClinicalTrials.gov: NCT06022406
Protocol version {3}	Protocol version 2.1, 11 November 2024
Funding {4}	Canadian Institutes of Health Research (CIHR) Canadian HIV Trials Network (CTN) (CTN PT038 grant to SI and JPR), the Canadian Cell Therapy Network (ThéCell), the Canadian Enterprise for HIV Cure (CanCURE 3.0)
Author details {5a}	Stéphane Isnard^1,2,3^, Carolina A. Berini^1,2^, Seema Nair Parvathy^4,5^, Hansen Feng^1^, Orthy Aiyana^1^, Léna Royston^1,2,3,6^, Tsoarello Mabanga^1,2^, Peter L. Lakatos^7^, Talat Bessissow^7^, Marina B. Klein^1,2,3^, Bertrand Lebouché^1,2,8,9^, Cecilia T. Costiniuk^1,2^, Bertrand Routy^10^, Michael S. Silverman^4,5^, Jean-Pierre Routy^1,2,11^ 1 Infectious Diseases and Immunity in Global Health Program, Research Institute, McGill University Health Centre, Montréal, QC, Canada 2 Department of Medicine, Division of Infectious diseases and Chronic Viral Illness Service, McGill University Health Centre, Montréal, QC, Canada 3 CIHR Canadian HIV Trials Network, Vancouver, BC, Canada 4 Department of Medicine, Division of Infectious Diseases, Western University, London, Ontario, Canada 5 Lawson Research Institute, St Joseph’s Health Care, London, Ontario, Canada 6 Division of Infectious Diseases, Geneva University Hospitals, Geneva, Switzerland 7 Division of Gastroenterology, McGill University Health Center, Montréal, QC, Canada 8 Centre for Outcomes Research and Evaluation, Research Institute of the McGill University Health Centre, Montréal, QC, Canada 9 Department of Family Medicine, McGill University Health Centre, Montréal, QC, Canada 10 Centre de Recherche du Centre Hospitalier de l’Université de Montréal, Montreal, QC, Canada 11 Division of Hematology, McGill University Health Centre, Montréal, QC, Canada
Name and contact information for the trial sponsor {5b}	Dr. Jean-Pierre Routy Jean-pierre.routy@mcgill.ca Chronic Viral Illness Service Royal Victoria Hospital 1001 Boulevard Décarie, Room D02.4017, Montreal, QC, H4A 3J1, Canada
Role of sponsor {5c}	As sponsor investigator of the Gutsy study, Dr. Jean-Pierre Routy oversees study design; data collection, management, analysis, and interpretation of data; writing of the report; and decides to submit the report for publication

### Introduction

#### Background and rationale {6a}

Antiretroviral therapy (ART) decreases human immunodeficiency virus (HIV) plasma viral load below the limit of detection, leading to major improvements in the health of people with HIV (PWH). However, the risk of developing inflammatory non-AIDS comorbidities such as cardiovascular diseases, fatty liver, neurocognition dysfunction, and cancer remains [1]. Factors associated with immune activation on ART include spontaneous cellular release of HIV RNA and proteins, cytomegalovirus (CMV) co-infection, and gut permeability allowing microbial translocation [2–5].

In PWH, an imbalanced gut microbiota composition is associated with gut epithelial damage, facilitating the translocation of bacterial and fungal products into the mucosa and bloodstream [6–9]. As it induces immune activation, microbial translocation has been linked with the development of non-AIDS comorbidities [4, 9–11]. Long-term ART only partially restores gut barrier function and fails to reduce inflammation to levels observed in uninfected controls. Hence, research focusing on improving the composition of the gut microbiota and the epithelial barrier function is important.

Furthermore, persistent HIV infection of long-lived memory CD4 T-cells and macrophages also contribute to immune activation and microbial translocation, creating a vicious cycle that nurtures inflammation [5, 12]. HIV reservoir produces RNA and protein that participate in immune activation and disruption of the immune functions [13, 14]. Importantly, the size of the HIV reservoir in CD4 T cells has been linked to the nadir CD4 T cell count, current CD4 T cell count, level of immune activation, and exhaustion of CD8+ T-cells [15–17]. It remains unknown whether the reduction of systemic inflammation can lead to a decrease in the size of the HIV reservoir in PWH.

#### Gut permeability and immune activation

HIV infection is characterized by functional impairment and CD4 T-cell decay. HIV preferentially replicates in the Th17 CD4 T-cells from the gut, inducing cell death and mucosal damage [18, 19]. Upon epithelial gut damage, microbial products penetrate the mucosa and pass into the bloodstream.

Markers of bacterial translocation, including lipopolysaccharide (LPS), LPS binding protein (LBP), and soluble CD14 (sCD14), have been correlated with immune activation and disease progression [10, 20, 21]. While bacterial translocation is thought to be a major cause of immune activation, we have shown that circulating β-D-glucan, a marker of fungal translocation, contributes to the immune activation in an LPS-independent manner [4, 9, 22].

On ART, epithelial gut damage and microbial translocation remains, contributing to a persistent systemic immune activation. Monocytes/macrophages, dendritic cells, and natural killer (NK) cells detect microbial products in the gut and draining mesenteric lymph nodes and secrete in response pro-inflammatory cytokines like interleukin 1β (IL-1β), IL-8, and tumor necrosis factor α (TNF-α). These cytokines drive CD4 T-cell activation, leading to elevated expression of the HIV co-receptor, C-C chemokine receptor (CCR) 5, and the gut homing receptor, CCR6 [19]. CD4 T-cells expressing CCR6 a cellular marker of their TH17 function have been shown by our group to be preferentially infected [19, 23].

#### Gut microbiota, dysbiosis, and immune regulation

The gut microbiota and its metabolites play a role in obesity, diabetes, cancer, and HIV infection. Their role includes food and metabolite processing, microbial ecological regulation, and immune regulation [24, 25]. Compared to uninfected controls, PWH present with relatively decreased abundance of Firmicutes and more abundant Proteobacteria in their gut microbiota, independently of sexual practice [6, 7]. The presence of *Lactobacilli* in stool is associated with higher CD4 T-cell count and a lower level of systemic immune activation [26]. Dysbiosis, an altered gut microbial composition, combined with microbial translocation has been linked to the risk of non-AIDS events and influences CD4 T-cell recovery on ART as reported by us and others [6, 9–11, 26]. Interestingly, gut microbiota composition has been linked with the size of circulating HIV reservoir in CD4 T-cells as well as with immune function, as illustrated by different microbiota in virologic/elite controllers as compared to progressors [27].

#### Summary of previous data with fecal microbiota transplant (FMT)

In PWH, the mucosal immune system, including gut protective Th17 cells, is lost within weeks post simian immunodeficiency virus (SIV) and HIV infection, contributing to damage of the barrier integrity, microbial translocation, and systemic immune activation [28, 29]. Fecal microbiota transplantation (FMT) of a healthy donor’s microbiota allows drastic changes in gut microbiota composition and has been suggested to attenuate dysbiosis, prevent gut barrier damage, and decrease immune activation [29, 30].

In a pilot study, Hensley-McBain et al. reported that FMT significantly increases the number of peripheral Th17 and Th22 cells and reduces CD4 T-cell activation in the gut of SIV-infected macaques receiving ART [31]. Moreover, the transplant was well tolerated, and no side effects were observed [31].

A small pilot study (*n* = 8) involving ART-treated individuals who received a single fecal microbiota transplantation (FMT) from a stool bank, administered via colonoscopy, reported no serious adverse effects during the 24-week follow-up period [32]. Microbial engraftment occurred but was partial and only limited to certain bacterial taxa. The authors considered that the limited effects of FMT might be related to the single dose of FMT and the delivery route which does not modify the small intestine microbiota. In Spain, Serrano-Villar et al. reported that repeated oral low-dose FMT in capsules was one way to safely introduce fecal microbiota from healthy donors to ART-treated PWH [33]. However, the study results showed that weekly low dose of oral FMT administered over 8 weeks in PWH on ART only induced a reduction of circulating levels of the gut damage marker I-FABP, observed solely 4 weeks after initiating FMT. Using proteomics, they later found a reduction in over 45 inflammatory proteins, which was sustained up to 16 weeks after the final FMT [34]. The study also did not assess HIV reservoir size. The authors suggested a higher dose of FMT could lead to a more pronounced physiological effect [33].

In patients with metastatic malignant melanoma, Routy B et al. utilized single large-dose FMT capsules prepared by Dr. Michael Silverman at Western University, London, ON, Canada. This approach was safe, well tolerated, and, for those having higher levels of microbial ingraftment, was asscociated with response to immunotherapy [35]. Based on encouraging results in oncology, we developped a similar approach to be used in PWH.

### Objectives {7}

The aim of this study is to assess the influence of large-dose FMT on gut permeability and immune activation in PWH receiving ART.

#### Primary endpoint and outcome measures

Changes in plasma levels of the robust gut permeability marker REG3α levels [36].

#### Secondary endpoint and outcome measures

1. Safety and tolerability of FMT measured by evaluating adverse events, hematology, and serum chemistries over the course of the study. These evaluations include HIV viral load, glucose levels, lipid profile, plasma levels of C-reactive protein (CRP), and D-dimer.
2. Changes in plasma levels of gut permeability markers including intestinal fatty acid binding protein (I-FABP) and LPS.
3. Changes in plasma levels of pro-inflammatory markers (1–3-β-D-glucan, IL-1β, IL-6, IL-8. IP-10, IL-17A and F, IL-22) and anti-inflammatory markers (IL-10).
4. Changes in T and myeloid cell activation in blood. CD4 and CD8+ T cell and monocyte activation levels will be measured by the expression of CD38 and HLA-DR and T cell exhaustion by PD-1.

#### Exploratory endpoint and outcome measures

1. Changes in microbiota composition and diversity in stools.
2. Changes in HIV reservoir size (proviral DNA and RNA) in blood CD4 T-cells.
3. Engraftment by comparing the gut microbiota of donors and participants.

#### Sub-study endpoint and outcome measures

1. Changes in inflammatory cytokines as well as T and myeloid cell activation in colon biopsies.
2. Changes in HIV reservoir size in colon CD4 T-cells.

### Trial design {8}

Trial CTN PT038 is an exploratory single-blind, randomized at a 1:1 ratio, placebo-controlled clinical trial to assess the influence of FMT on microbial translocation, immune activation, and HIV reservoir in PWH on ART.

## Methods: participants, interventions, and outcomes

### Study setting {9}

The Gutsy study is a single-site, academic hospital-based clinical trial. All study activities are being conducted at Chronic Viral Illness Service (CVIS), McGill University Health Centre—Glen Site, Montreal, Quebec, Canada.

This academic medical center provides comprehensive care to more than 2000 PWH, offering a robust infrastructure for participant recruitment and study execution.

### Eligibility criteria {10}

#### Inclusion criteria

Participants are eligible for the study if they meet the following inclusion criteria:

1. Male or female adults ≥ 18 years of age.
2. Documented HIV-1 infection by western blot, enzyme immunoassay (EIA), or viral load assay.
3. On ART for at least 3 years, and stable ART regimen (same prescription) for at least 3 months.
4. Undetectable viral load < 50 copies/mL for the past 3 years. Viral blips below 200 copies/mL are allowed if preceded and followed by a HIV viremia below 50 copies/mL.
5. CD4 count greater than 200 cells/µL and a CD4/CD8 ratio below 1 to select people with higher risks of inflammatory non-AIDS comorbidities and dysbiosis.
6. Able to communicate adequately in either French or English.
7. Able and willing to provide written informed consent prior to screening.
8. Women of childbearing potential must have a negative serum pregnancy test at screening.
9. Women of childbearing potential must agree to use one of the study-approved methods of birth control while in the study and until 2 weeks after completion of the study.
10. Women of non-childbearing potential as defined as either post-menopausal (12 months of spontaneous amenorrhea and ≥45 years of age) or physically incapable of becoming pregnant with documented tubal ligation, hysterectomy, or bilateral oophorectomy.
11. Sexually active men with a female partner of childbearing potential must agree to one of the study-approved methods of birth control until 2 weeks after study completion.

#### Exclusion criteria

Participants are not eligible to participate in the study if they meet any of the following exclusion criteria:

1. Known allergy/hypersensitivity to polyethylene glycol.
2. Current AIDS-related event or serious health condition including systemic infections in the last 3 months.
3. Severe systemic diseases (e.g., uncontrolled hypertension, chronic renal failure) or active uncontrolled infections.
4. Co-infection with active hepatitis B or C virus.
5. Current use or have used in the past 3 months: immune-modulatory agents, antibiotics, or morphine as these drugs modulate gut microbiota.
6. Diagnosis of diabetes mellitus (HbA1c ≥ 6.5%) as defined by the Canadian Clinical Practice Guidelines for the Prevention and Management of Diabetes.
7. Recent changes in dietary habits, intermittent fasting, chronic constipation, or laxative use as these can affect gut microbiota composition.
8. Psychiatric or cognitive disturbance or any illness that could preclude compliance with the study.
9. Current participation in an experimental therapy study or receipt of experimental therapy within the last 6 months.
10. Women who are planning to become or who are pregnant, or breast-feeding.
11. A score of higher than 8 on the Alcohol Use Disorders Identification Test (AUDIT) questionnaire at the screening visit, suggesting an alcohol abuse problem [37, 38].

### Who will take informed consent? {26a}

Study staff under the supervision of the qualified investigator (Dr. Jean-Pierre Routy) will be responsible for obtaining informed consent from potential participants. Consent will be obtained during the screening visit (visit 1). Study staff will explain the study procedures, risks, and benefits in either French or English. The main study consent form will be reviewed with the participant. If the participant agrees, they will be asked to sign the informed consent document before any study procedures begin. As for the optional sub-study (colon mucosal biopsies), a separate informed consent form will be explained and must be signed by participants who wish to join this part of the study. Participants must first consent to the main study before being offered the optional sub-study.

### Additional consent provisions for collection and use of participant data and biological specimens {26b}

Participants are informed that stored research specimens may be used for future testing, should new markers or technologies become available. Any unforeseen or additional tests aligned with the study’s purpose (e.g., new methods for assessing gut health or HIV reservoirs) may be conducted on these samples. These future uses are covered under the initial informed consent, which specifies the scope of potential analyses.

## Interventions

### Explanation for the choice of comparators {6b}

The inclusion of a placebo group is essential to establish rigorous control. This control group allows for the evaluation of the true effect of FMT on gut permeability, immune activation, and HIV reservoir size, thereby minimizing any potential bias. By comparing the results of the FMT group with the placebo group, the specific impact of the treatment is determined more accurately.

The integrity of the blinding is ensured by making the placebo capsules identical in appearance and weight to the FMT capsules. This physical similarity is crucial so that participants cannot distinguish between the active treatment and the placebo, thus avoiding any subjective influence on their responses.

The choice of a placebo comparator is based on the need to isolate the effects of the FMT and control for the placebo effect, especially considering the subjective nature of some outcome measures, such as gastrointestinal symptoms. Microcrystalline cellulose is specifically selected because it is an inert substance that does not interact with the gut microbiota or the immune system. This ensures that any changes observed in the FMT group can be attributed to the treatment itself, and not to the substance used in the placebo.

### Intervention description {11a}

**Fig. 1:**
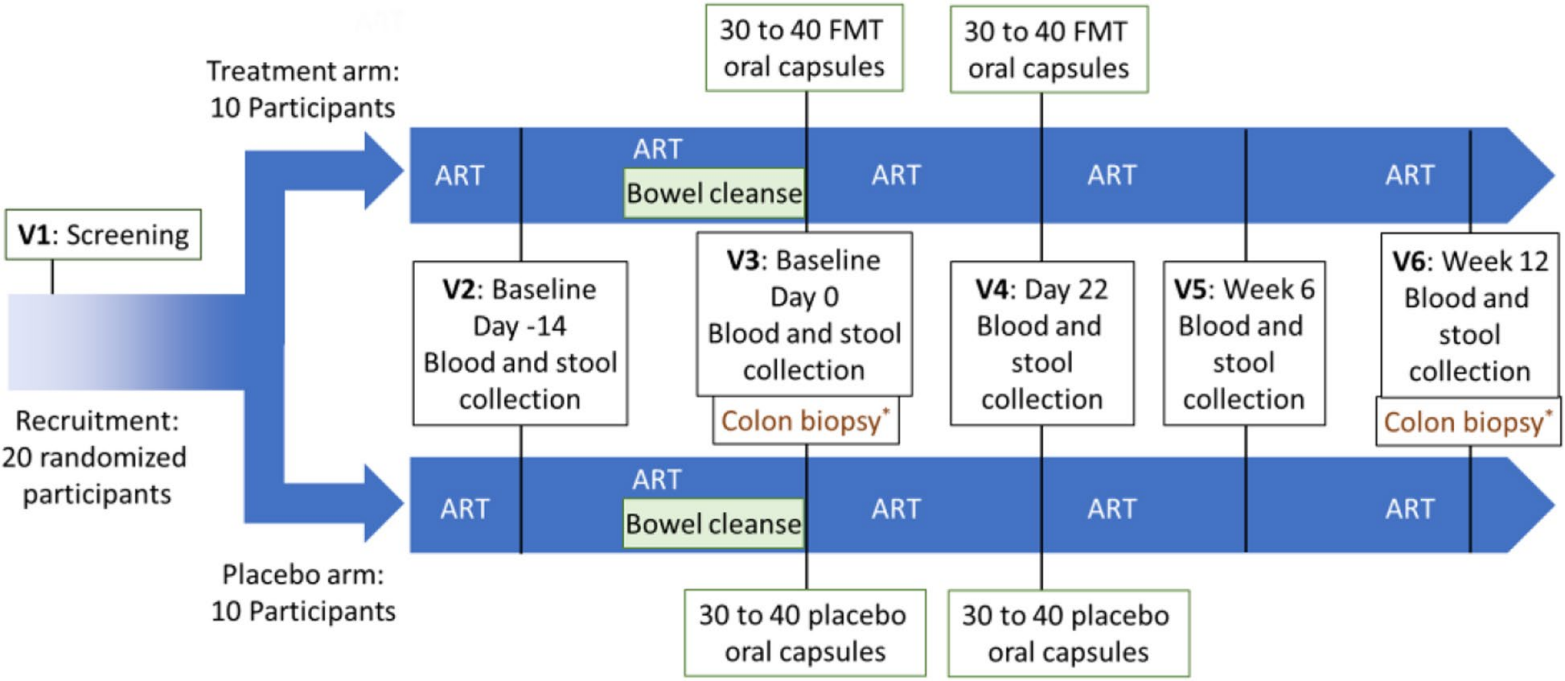
Flow chart. Visit 1, the screening visit, will take place up to 7 weeks prior to the baseline 1 visit (day −14 ± 7 days, visit 2). At the screening visit, the informed consent document will be explained to the participant and will be signed prior to any screening and study activities. Two baseline visits will be conducted, at day −14 and day 0. All study visits will be relative to baseline 2 (day 0, visit 3). Data collected at these two baseline visits will be directly compared to determine intra-patient variability. A bowel cleanse will be given to participants prior to the treatment visit (visit 3). After the collection of blood and stool samples, two FMT treatment or placebo (30 to 40 capsules each) will be given to participants at day 0 (visit 3) and day 22 (visit 4). Blood and stool will be collected post-treatment at week 6 and week 12 (visit 5 and visit 6). Schedule of events is described in Supplementary Table 1. *Optional gut biopsies will be taken for the sub-study at the indicated time points. The consent form for the optional gut biopsy will also be explained at the screening visit, but consent for this will not be necessary to be part of the main study. Randomization will be conducted after screening one eligibility is confirmed to allow time to recruit stool donors and prepare FMT capsules for those in the FMT arm

Participants are asked to continue using their typical diet throughout the study. At a first baseline visit, blood and stools are collected. Study participants then undergo a polyethylene glycol-based and electrolyte bowel cleanse the day before baseline 2 visit and then receive two doses of FMT or placebo taken orally in capsules 22 days apart. Follow-up visits are scheduled post-intervention 3 and 9 weeks after FMT/placebo treatment (see schedule of events (Fig. 1) and Supplementary Table 1 describing the schedule of events).

In an optional sub-study evaluating gut mucosa architecture, immune activation, and reservoir size in the gut, colon biopsies are collected before the first dose (baseline 2) and at the end of the study, 9 weeks after the 2nd dose of FMT or placebo (week 12).

#### Interventional product: FMT and placebo capsules

The study product consists of FMT capsules prepared by Dr. Silverman’s team in London, Ontario, Canada. Dr. Silverman has received approval from Health Canada to prepare FMT and placebo capsules for the Gutsy study (Control #292512).

Thirty to forty capsules, depending on the fiber content of the donor’s feces, are prepared from 80 to 100 g of healthy human feces from a single healthy donor, encapsulated in triple layer gelatin capsules. A total of 30 to 40 capsules are administered as a single dose. Capsules are prepared following a modified method as described by Kao et al. and Dr. Silverman’s group [30, 35, 39–43]. Capsules are shipped to Montreal on dry ice and stored at −80 °C until use. Capsules from the same donors are given at visits 3 and 4.

Placebo capsules contain microcrystalline cellulose for equivalence in weight and color, also encapsulated in triple layer gelatin capsules. Placebo capsules are shipped and stored at room temperature until dispensation.

### Criteria for discontinuing or modifying allocated interventions {11b}

Discontinuation or modification of the allocated interventions may occur in the following situations: vomiting during capsule intake, adverse events (AEs), abnormal laboratory findings, and investigator judgment.

Vomiting during capsule intake: If a participant vomits during or shortly after taking the FMT or placebo capsules, the investigator will evaluate the reason and a decision will be made to either repeat dosing or exclude the participant from further treatment.

Adverse events (AEs): If a participant experiences persistent or clinically significant adverse events after the first dose, the second dose will not be administered. The participant will continue follow-up visits to monitor and resolve the AE, but no further study product will be given.

Abnormal laboratory findings: If abnormal lab results persist following the first dose, the second dose will not be administered. The participant will be referred for appropriate medical follow-up.

Investigator judgment: The investigator may discontinue the intervention at any point based on clinical judgment regarding safety.

### Strategies to improve adherence to interventions {11c}

To ensure the integrity of the study, adherence to both the fecal microbiota transplantation (FMT) or placebo regimen and antiretroviral therapy (ART) will be rigorously monitored by:

- Direct observation of capsule intake: All participants take the FMT or placebo capsules under direct supervision by trained study staff at the study site. This ensures complete adherence to dosing on both treatment days (day 0 and day 22).
- Ongoing communication and counseling: Participants are educated about the importance of adherence to both study procedures and their ongoing ART. Study staff follow up and maintain communication with participants throughout the study.
- Scheduling support and reminders: Participants are provided with clear scheduling, instructions, and reminders for upcoming visits and stool/blood collection.

### Relevant concomitant care permitted or prohibited during the trial {11d}

#### Permitted concomitant care and medications

- All participants must continue their ART throughout the entire study period.
- Concomitant medications, supplements, natural remedies, and foods are generally permitted except those explicitly listed in the exclusion criteria.
- Use of non-prescription drugs is allowed if approved by the investigator and must be documented.

#### Prohibited or restricted concomitant care and interventions

- Excluded at screening (and thus effectively prohibited during the trial):
  - Use within the past 3 months of antibiotics, prophylactic antibiotics, immune-modulatory agents, and morphine. These substances are excluded due to their effects on gut microbiota.
  - Recent changes in dietary habits, intermittent fasting, chronic constipation, or laxative use. These can significantly affect gut microbiota and interfere with outcomes.
  - Current participation in an experimental therapy study or receipt of such therapy in the last 6 months.
- During the study:
  - Participants are asked to refrain from consuming non-prescription drugs for 48 h prior to each study visit, unless pre-approved by the investigator.
  - Alcohol use should be limited to a maximum of one alcoholic drink per day, preferably less than daily as alcohol negatively modulates gut microbiota and increases inflammation.
  - Participants should refrain from cannabis and limit alcohol (no more than one drink with dinner) in the 24 h prior to each study visit.
  - Street drug use and cigarette smoking: not outright prohibited, but surveyed at each visit and may influence continued participation based on investigator’s judgment.
- At investigator discretion:
  - Any new medication or procedure must be reported.
  - The investigator evaluates whether it may interfere with study interpretation and may lead to withdrawal if necessary.

### Provisions for post-trial care {30}

FMT capsules will not be provided at the end of the study, as clinical benefits will have not yet been demonstrated. This means participants will not continue receiving FMT post-study, even if they benefited from it during the trial.

Participants who experience unresolved clinically significant AEs will be followed until resolution or stabilization. Monthly follow-ups may occur until resolution, and these will be documented in the electronic case report form (eCRF). Unscheduled visits can be scheduled as needed to monitor and manage AEs. If needed, participants will be referred to appropriate specialists. If a participant discontinues the study due to an AE, the investigator is responsible for their clinical follow-up and safety follow-up visits will be arranged, and participants may be referred for further care.

The study is conducted in accordance with ICH-GCP and Health Canada regulations, which requires reporting and medical management of harms. Should the participant suffer any harm following administration of the study drug or any other procedure related to the research study, he or she will receive all the care and services required by his or her state of health.

### Outcomes {12}

Table 1 is a structured summary of the primary, secondary, and exploratory outcomes, including the measurement variables, analysis metrics, methods of aggregation, time points, and clinical relevance.

**Table 1:** Study outcome details

a. Primary outcome					
Outcome	Details				
Measurement variable	Plasma levels of REG3α (a marker of gut permeability)				
Analysis metric	Change from baseline compared between FMT and placebo groups				
Method of aggregation	Mean or median (analyzed using *t*-test or Wilcoxon rank sum test)				
Time points	Baseline (visits 2 and 3), week 6, and week 12				
Clinical relevance	REG3α is a key biomarker for gut epithelial damage and microbial translocation. Reducing REG3α suggests improved gut barrier integrity, which may decrease chronic immune activation in ART-treated PWH				
b. Secondary outcomes					
Outcome	Measurement variable(s)	Analysis metric	Method of aggregation	Time points	Clinical relevance
Safety and tolerability of FMT	Adverse events, hematology, serum chemistries (e.g., ALT, AST, glucose, lipids, hs-CRP, D-dimer)	Incidence/frequency; change from baseline, compared between FMT and placebo arms	Proportion; mean/median	Throughout study (all visits)	Evaluates whether FMT is safe and well tolerated in PWH
Markers of gut permeability	Plasma I-FABP, LPS, β-D-glucan	Change from baseline, compared between FMT and placebo arms	Mean/median (Multiplex, ELISA, Fungitell assay)	Baseline, week 6, week 12	I-FABP, LPS, and β-D-glucan are established indicators of gut epithelial damage and bacterial and fungal translocation
Inflammatory/anti-inflammatory cytokines	IL-1β, IL-6, IL-8, IL-17A/F, IL-22, IL-10, IL-37, IP-10	Change from baseline, compared between FMT and placebo arms	Mean/median (Multiplex or ELISA)		Evaluates whether FMT could reduce immune activation and decrease the risk of non-AIDS comorbidities
Immune cell activation	Flow cytometry of HLA-DR, CD38 on T-cells, CD83, CD86 on myeloid cells	Change from baseline, compared between FMT and placebo arms	Mean/median		
c. Exploratory outcomes					
Outcome	Measurement variable(s)	Analysis metric	Method of aggregation	Time points	Clinical relevance
Microbiota composition and diversity	16S, 18S rDNA sequencing, metagenomics in stool samples	Change from baseline, compared between FMT and placebo arms	Alpha/beta diversity metrics; LEfSe; DESeq2	Baseline, week 6, week 12	Identifies changes in microbiota profile
HIV reservoir size (blood and gut)	Quantitative PCR on sorted CD4 T cells	Fold change from baseline, compared between FMT and placebo arms	Mean/median	Baseline, week 12	Potential for reducing latent HIV reservoir, supporting cure strategies
Engraftment analysis	Comparison of donor and recipient microbiota (16S/18S/metagenomics)	Similarity analysis	Proportion/shared taxa	All fecal sample time points	Measures the success of microbial transplantation
d. Sub-study outcomes (optional colon biopsies)					
Outcome	Measurement variable(s)	Analysis metric	Method of aggregation	Time points	Clinical relevance
Gut mucosa architecture	Immunostaining for claudin-3, occludin	Comparison pre-/post-intervention, compared between FMT and placebo arms	Microscopic evaluation	Baseline 2, week 12	Evaluates epithelial integrity, a key factor in preventing microbial translocation
Mucosal immune activation	Flow cytometry on colon biopsy cells (CD38, HLA-DR on T-cells, CD83, CD86 on myeloid cells); qPCR for cytokines	Change from baseline, compared between FMT and placebo arms	Mean/median		Measures direct effects of FMT on gut immune environment
HIV reservoir in gut CD4 T cells	qPCR on CD4 T cells from biopsies	Fold change, compared between FMT and placebo arms	Mean/median		Assesses FMT’s effect on mucosal HIV persistence

The primary endpoint for analysis will be at weeks 6 and 12.

The efficacy outcomes (REG3α, cytokines, immune activation) assess whether FMT can reduce chronic immune activation, enhance gut integrity, and potentially impact the HIV reservoir, all of which are key to improving long-term health in PWH.

On the other hand, the harm outcomes (safety, tolerability) ensure that any potential risks associated with FMT are detected and managed promptly, particularly given the immune status of participants.

### Participant timeline {13}

The study flow chart shows the time schedule of enrollment, interventions, assessments, and visits for participants.

### Sample size {14}

A total of 20 PWH participants will be enrolled, randomized 1:1 into two groups: FMT group (10 participants) and placebo group (10 participants).

The influence of gut microbiota targeting therapies on REG3α levels has not been tested yet. Hence, we calculated that based on the difference in REG3α levels between ART-treated (2441 ± 630 pg/mL) and HIV-negative controls (715 ± 243 pg/mL) [36], a sample size of 5 participants per group would allow 80% power to observe an effect, with a *p* value of 5% (https://clincalc.com/stats/samplesize.aspx). The clinical assumption and hypothesis is that complete change in the gut microbiota towards the one of HIV-negative controls would lower REG3α levels to those of controls. However, as previous FMT trials did not obtain 100% engraftment of the donor microbiota, sample size per group was doubled with 10 participants per arm. Due to the exploratory nature of the study, the small sample size is considered sufficient to detect preliminary signals in biomarkers of gut permeability, immune activation, and microbial translocation. Our study will inform larger, future randomized controlled trials as the variability in outcomes like REG3α, inflammatory markers, and microbiota composition will be estimated to support future power calculations.

As for the sub-study sample size, the optional colon biopsy sub-study includes a subset of the main study participants. No specific target number for this subgroup is defined, but participants must first qualify for and consent to the main study. In previous trials, around 50% of participants agreed to partake in the sub-study with colonoscopy as it can benefit participant with cancer screening [44–46].

### Recruitment {15}

Some of the recruitment strategies to achieve the target sample size of 20 participants are outlined below:

- In-clinic recruitment by study team: The qualified investigator (Dr. Routy) and study staff will identify eligible participants directly from the clinical HIV cohort at the CVIS. This clinic provides care to more than 2000 PWH, offering a robust recruitment base.
- Direct outreach to potential participants: Study staff will explain the study and its procedures to prospective participants during clinic visits. They will review inclusion and exclusion criteria and discuss the voluntary nature of participation.
- Promotional activities by investigators: Teleconferences and in-person meetings will be held with the research team to coordinate and encourage recruitment. The sponsor investigator and qualified investigator will promote the study at local medical meetings and HIV conferences (e.g., CTN semi-annual meetings). Study has been advertised by the Montreal LGBTQ journal *Fugues*. A quick video explaining the study rationale was prepared (https://www.ctnplus.ca/study/ctnpt-038-the-gutsy-study/).

## Assignment of interventions: allocation

### Sequence generation {16a}

Participants are assigned to one of two arms: FMT capsules (group 1) or placebo capsules (group 2) allocated with 1:1 ratio.

#### Method of randomization

A statistician developed the randomization list prior to study commencement. A statistician will generate a list of random allocations in blocks using the PLAN procedure in Statistical Analysis System (SAS) software. The generated allocation sequence sequentially included in numbered sealed envelopes containing the group assignment. The number of placebo capsules, ranging from 30 to 40 per dose, is randomly selected based a uniform distribution using the RANUNI Function.

#### Blinding design

The study is single-blind so participants are blinded to the group assignment. Study staff who enroll participants are not blinded, but the sequence is concealed until after participant eligibility is confirmed.

### Concealment mechanism {16b}

The allocation sequence is implemented using sequentially numbered, sealed envelopes. These envelopes are prepared in advance by the CIHR/CTN statistician. Each envelope contains the randomization group assignment (FMT or placebo). Envelopes are opened only after the participant is screened and confirmed eligible for the study ensuring the allocation sequence is concealed from enrolling staff until the point of assignment.

### Implementation {16c}

A statistician designated by the study team generated the randomization list before study initiation. Study staff under the supervision of the qualified investigator (Dr. Jean-Pierre Routy) at the McGill University Health Centre (MUHC), Glen Site, is in charge of enrolling participants. Once enrolled, study staff open the next sequentially numbered, sealed envelope after confirming participant eligibility to determine group assignment (FMT or placebo). The randomization group is recorded in the electronic case report form (eCRF) after envelope opening.

## Assignment of interventions: blinding

### Who will be blinded {17a}

Table 2 shows a summary of blinding assignment.

**Table 2:** Summary of blinding assignment

Blinded role	Blinded?	How blinding is maintained
Trial participants	Yes	Participants are not told whether they are receiving FMT or placebo capsules. Both capsule types are identical in appearance, weight, and color
Care providers	No	Study staff administering capsules are aware of group assignments after opening randomization envelopes
Outcome assessors	No	Not explicitly stated; however, since the same staff may conduct follow-up, they may not be blinded
Data analysts	No	No indication in the protocol that data analysts will be blinded

### Procedure for unblinding if needed {17b}

No routine unblinding is planned, as the single-blind design only keeps participants unaware of their group assignment. Since the study staff are not blinded, they are aware of the intervention assignment and can access this information when medically necessary. The protocol emphasizes participant safety, which implies unblinding is allowed if it serves clinical care.

## Data collection and management

### Plans for assessment and collection of outcomes {18a}

Baseline data include medical and medication history, physical examination, and laboratory parameters such as hematology, biochemistry, HIV viral load, CD4/CD8 counts, and serology. Study outcomes will be assessed through clinical evaluation, laboratory assays, and validated research procedures at multiple time points across six study visits.

#### Outcome measures and instruments

- Primary outcome: Changes in REG3α plasma levels (a validated marker of gut permeability) using ELISA.
- Secondary outcomes: Changes in inflammatory and gut permeability markers (e.g., I-FABP, LPS, β-D-glucan, cytokines) via ELISA or Multiplex assays, and immune activation markers via flow cytometry (HLA-DR, CD38, CD83, CD86).
- Exploratory outcomes: Microbiota composition via 16S rDNA sequencing and HIV reservoir size by qPCR.
- Sub-study measures: Gut architecture and immune activation assessed through paraffin-embedded biopsy immunostaining (e.g., claudin-3/occludin) and flow cytometry.

All instruments and assays have been validated and previously published by the team or others in peer-reviewed literature. Laboratory methods adhere to standard operating procedures (SOPs) including those from the International Human Microbiota Standards (IHMS).

#### Data quality assurance

- All study personnel will be trained in good clinical practice (GCP) and study-specific procedures.
- Sample collection, processing, and storage follow strict protocols to preserve sample integrity.
- eCRFs will be used for data entry, with built-in validation rules and range checks.
- Duplicate testing will be performed for critical assays when feasible (e.g., ELISA replicates).
- Flow cytometry and sequencing assays will be performed by qualified laboratory personnel using validated gating and bioinformatics pipelines.

#### Source documentation and access

Source documents include medical charts, lab results, and signed consent forms. eCRFs will be considered the official data repository. The database will be managed under MUHC’s data security policies, with restricted access and audit trails.

#### Reference to data collection forms

All data collection forms (e.g., questionnaires, lab requisitions, eCRF templates) are maintained separately in the study’s operations manual and are available upon request but are not included in the protocol document.

### Plans to promote participant retention and complete follow-up {18b}

Participant retention and study completion are essential to the validity of the trial outcomes. The following strategies will be implemented to promote participant adherence and minimize loss to follow-up.

#### Retention strategies

- Clear communication: Participants will receive detailed written and verbal instructions regarding study procedures, visit schedules, and the importance of completing all follow-up visits.
- Dedicated study staff: Participants will be followed closely by a consistent team of research coordinators, nurses, and investigators to foster trust and engagement.
- Appointment reminders: Reminders via phone, email, or text message will be provided prior to each study visit.
- Flexible scheduling: Study visits will be scheduled at convenient times for participants whenever possible, including early mornings or late afternoons.
- Participant engagement: A positive study experience will be encouraged through consistent communication, respectful interactions, and regular updates on study progress and findings.
- Incentives: Modest, ethically appropriate compensation will be provided for time and inconvenience, in accordance with REB-approved policies.

#### Follow-up and data collection for early withdrawals

If a participant discontinues or deviates from the assigned intervention, every effort will be made to retain them for follow-up assessments. This includes:

- Scheduling remaining study visits even if the participant is no longer receiving the study intervention.
- Documenting reasons for withdrawal or protocol deviations in the eCRF.
  o Collecting available clinical and laboratory outcome data at the time of withdrawal, including adverse events, vital signs, HIV viral load and CD4/CD8 counts, blood and stool samples for biomarker analysis (when feasible), and questionnaire data (e.g., AUDIT-C, symptom checklists)

#### Data inclusion

Participants who deviate from the protocol or discontinue early will still be included in the intention-to-treat analysis when appropriate. Efforts will be made to obtain final visit data to ensure robust outcome assessment and minimize missing data.

### Data management {19}

#### Data entry and coding

Data for this study will be recorded using eCRFs on the InForm platform, hosted on a secure, Health Canada-compliant clinical data management system. All study data will be entered into the system by trained research staff. Each participant will be assigned a unique study identifier to ensure confidentiality and facilitate accurate tracking.

- Source documents will include clinical charts, lab reports, and signed informed consent forms.
- Data will be entered from source documents into the eCRF as soon as possible following each study visit.
- All laboratory results will be coded using standardized variable names aligned with CDISC/MedDRA conventions where applicable.

#### Data quality control

To ensure high data quality and integrity, the following quality assurance procedures will be implemented:

- Automated range checks: The eCRF system will include validation rules and range checks to flag missing, inconsistent, or out-of-range values at the time of data entry.
- Query management: Data queries generated by the system or monitors will be addressed and resolved by site staff in a timely manner.
- Monitoring: Regular internal monitoring will be conducted to verify data against source documents. External monitoring will be performed in accordance with the study’s monitoring plan.

#### Data security and storage

All electronic data will be stored on password-protected servers compliant with institutional and regulatory data privacy standards. Access to the eCRF system will be role-based and restricted to authorized personnel. Data will be backed up regularly, and audit trails will be maintained to track all changes.

- Paper documents (e.g., consent forms, backup logs) will be stored in locked filing cabinets in a secure research office at the MUHC.
- Biological sample data will be linked to study IDs only, with no directly identifiable information attached.

#### Data retention and archiving

Data will be retained for a minimum of 25 years post-study completion, in accordance with Health Canada and institutional guidelines. At the end of the retention period, data will be securely archived or destroyed as per SOPs.

#### Reference to additional procedures

A detailed Data Management Plan (DMP), including system specifications, user access logs, SOPs, and data cleaning workflows, is maintained separately and is available upon request from the study investigator.

### Confidentiality {27}

Personal information of all potential and enrolled participants will be collected, managed, and stored with strict adherence to privacy regulations and institutional policies to ensure confidentiality is protected at all stages of the study—before, during, and after participation.

#### Collection and storage of personal information

- Identifiable information (e.g., name, date of birth, health card number) will be collected solely for the purpose of screening, enrollment, and follow-up.
- Upon enrollment, each participant will be assigned a unique study ID. This ID will be used on all case report forms (CRFs), laboratory samples, and stored data.
- Personal identifiers will be stored separately from study data in a secure, access-restricted location at the McGill University Health Centre (MUHC).

#### Access and sharing

- Only authorized members of the study team, including the principal investigator, research coordinators, and relevant regulatory monitors, will have access to identifiable participant information.
- Data shared with collaborators, laboratories, or external monitors will be de-identified and labeled with study IDs only.
- Any transfer of data outside the study site will follow data sharing agreements and comply with applicable privacy laws (e.g., Canadian Personal Health Information Protection Acts, GCP guidelines).

#### During and after the study

- Identifiable information will not appear in any reports, presentations, or publications.
- All electronic data will be stored on encrypted, password-protected servers, with role-based access controls and full audit trails.
- Paper files will be stored in locked cabinets in secure areas with limited access.
- Confidentiality obligations apply indefinitely, even after the trial has ended.

#### Participant notification

Participants will be informed of these confidentiality protections in the informed consent process. They will also be told how their data will be used, how it will be protected, and that withdrawal from the study does not affect their rights to medical care or confidentiality.

### Plans for collection, laboratory evaluation, and storage of biological specimens for genetic or molecular analysis in this trial/future use {33}

Biological specimens including blood, stool, and optional colon mucosal biopsies will be collected at designated study visits for both clinical and research purposes, including molecular and immunologic analyses.

- Blood samples: Collected at visits 2, 3, 4, 5, and 6 (~60 mL per visit) for isolation of plasma, serum, and peripheral blood mononuclear cells (PBMCs).
  - Plasma/serum will be analyzed for inflammatory markers, gut permeability biomarkers (e.g., REG3α, I-FABP, LPS, BDG), and cytokine profiles using ELISA and Multiplex.
  - PBMCs will be analyzed by flow cytometry (e.g., CD4/CD8 T cell activation, PD-1 expression) and used for HIV reservoir quantification by nested qPCR and Tat/Rev Induced Limiting Dilution Assay (TILDA).
- Stool samples: Collected at visits 2 through 6 for microbiota analysis.
  - DNA will be extracted and analyzed using 16S and 18S rDNA sequencing and metagenomic techniques to assess microbial composition, diversity, and donor engraftment.
- Colon mucosal biopsies (optional sub-study): Collected at visits 3 and 6 (for consenting participants).
  - Biopsies will be fixed or frozen for immunohistochemistry (e.g., claudin-3/occludin, myeloperoxidase staining), immune cell isolation, and HIV reservoir quantification via qPCR or RNAscope.

All laboratory analyses will be performed by qualified personnel at the MUHC Research Institute and collaborating labs, using validated and standardized protocols.

#### Future use and storage

With participant consent, leftover biological specimens will be stored for potential future research on emerging biomarkers of gut permeability or immune activation, new methods for HIV reservoir quantification, advanced microbiome, or transcriptomic profiling.

Stored specimens will be de-identified and linked only to a study ID, frozen at −80 °C or in liquid nitrogen, depending on sample type and securely stored at the MUHC research laboratories under the supervision of the principal investigator.

Any future research using these specimens will require approval by an appropriate Research Ethics Board (REB) and must comply with applicable privacy laws and institutional policies.

Participants may opt in or out of specimen storage for future use during the informed consent process.

## Statistical methods

### Statistical methods for primary and secondary outcomes {20a}

#### Primary outcome analysis

The primary outcome, change in plasma REG3α levels (a marker of gut permeability), will be analyzed using a within-subject comparison (pre- (baselines 1 and 2) vs. post-intervention (weeks 6 and 12)) and between-group comparison (FMT vs. placebo).

Descriptive statistics will be used to summarize REG3α levels at each time point. Paired *t*-tests or Wilcoxon signed-rank tests will assess within-group changes over time, based on data distribution. Between-group differences in change from baseline will be assessed using independent *t*-tests or Mann-Whitney *U* tests. A 95% confidence interval for the mean difference will be reported.

#### Secondary outcome analysis

Secondary outcomes include changes in inflammatory and gut permeability markers (e.g., I-FABP, LPS, cytokines), immune activation markers (e.g., HLA-DR, CD38 on T cells and CD83, CD86 on myeloid cells), and safety and tolerability outcomes (e.g., adverse events, hematology, HIV viral load).

These will be analyzed using repeated measures ANOVA or mixed-effects models to evaluate group-by-time interactions, non-parametric tests where assumptions are violated, and categorical variables (e.g., adverse event incidence) analyzed using chi-square or Fisher’s exact test.

All tests will be two-sided with a significance level set at *α* = 0.05.

#### Additional analyses

Exploratory endpoints, such as microbiota diversity, will be analyzed using multivariate methods (e.g., LEfSe, DESeq2).

#### Reference to statistical analysis plan

Further details on statistical procedures, variable definitions, and subgroup analyses are described in the Statistical Analysis Plan (SAP) maintained separately from the protocol. The SAP will be finalized prior to database lock and is available upon request.

### Interim analyses {21b}

No formal interim efficacy analyses are planned. However, interim safety monitoring will be conducted regularly to assess for any unexpected adverse events or safety concerns related to the study intervention (FMT or placebo).

Descriptive safety data (e.g., frequency and severity of adverse events, lab abnormalities) will be summarized after the first 5 and 10 participants have completed both treatment visits and at least one follow-up; this safety monitoring will focus on tolerability of FMT, consistency of laboratory findings, and any serious or unexpected adverse events.

#### Stopping guidelines

The study may be paused or terminated early based on the occurrence of unexpected serious adverse events (SAEs) potentially related to the intervention or emerging data from this or external studies that indicate unacceptable risk or lack of feasibility.

Stopping decisions will consider the nature, frequency, and severity of adverse events and the recommendations from the Data Safety Monitoring Committee (DSMC).

Only the DSMC and the sponsor investigator (Dr. Jean-Pierre Routy) will have access to cumulative interim safety data. The DSMC will review unblinded safety data and provide recommendations regarding study continuation, modification, or termination. Final decisions regarding early termination of the trial will rest with the sponsor investigator in consultation with the DSMC and REB, based on participant safety and scientific justification.

### Methods for additional analyses (e.g., subgroup analyses) {20b}

#### Subgroup analyses

Subgroup analyses will be exploratory and performed to assess whether the treatment effect varies by specific baseline characteristics. Potential subgroups include:

- CD4/CD8 ratio (e.g., ≤0.5 vs. >0.5)
- Age groups (e.g., <50 vs. ≥50 years)
- Sex at birth
- Baseline microbiota composition or diversity metrics
- Presence or absence of optional colon biopsy participation

Subgroup comparisons will use interaction terms in regression models or stratified analyses to explore differential responses to FMT vs. placebo. These analyses are hypothesis-generating and will be interpreted with caution due to limited power.

#### Adjusted analyses

If relevant baseline imbalances are observed between the intervention groups, adjusted analyses may be conducted using multivariable linear or logistic regression models to control for potential confounders (e.g., age, baseline inflammatory marker levels, ART regimen duration).

#### Statistical considerations

All additional analyses will be clearly labeled as exploratory. Results will be interpreted in the context of the small sample size and multiple comparisons and no formal correction for multiplicity will be applied in these exploratory analyses.

### Methods in analysis to handle protocol non-adherence and any statistical methods to handle missing data {20c}

#### Analysis populations

- Intent-to-treat (ITT) population: All randomized participants will be included in the primary and secondary outcome analyses according to their originally assigned intervention group, regardless of adherence or protocol deviations. This approach preserves the benefits of randomization and reflects real-world effectiveness.
- Per-protocol (PP) population: A secondary analysis will be conducted including only those participants who received both doses of the assigned intervention and completed the study without major protocol deviations. This will help assess the efficacy of the intervention under ideal conditions.

#### Handling protocol non-adherence

Participants who discontinue the intervention early or deviate from the protocol (e.g., missed doses, skipped visits) will still be followed for outcome data collection when possible. These participants will remain in the ITT analysis. Sensitivity analyses may compare results from ITT and PP populations to assess the robustness of findings.

#### Handling missing data

- Descriptive analysis: The extent, reasons, and patterns of missing data will be summarized.
- Imputation strategies:
  o If missing data are minimal and assumed to be missing completely at random (MCAR), a complete-case analysis may be performed.
  o If appropriate, multiple imputations will be used to address missing data under the assumption of missing at random (MAR). Imputation models will include treatment group, baseline values, and other relevant covariates.
  o For repeated measures, mixed-effects models will be used, which can accommodate some missing data under the MAR assumption without imputation.
- Sensitivity analyses: Additional sensitivity analyses may be performed to assess the impact of different assumptions about missingness (e.g., best-case/worst-case scenarios).

All methods used for handling missing data will be described in detail in the SAP.

### Plans to give access to the full protocol, participant-level data, and statistical code {31c}

There are no plans to publicly share the full protocol, participant-level dataset, or statistical code except in the present publication. Access to these materials may be considered on a case-by-case basis upon request, in accordance with institutional policies, participant confidentiality agreements, and applicable regulatory and ethical guidelines. Requests should be directed to the sponsor investigator, Dr. Jean-Pierre Routy, and will require appropriate data use agreements and REB approval.

## Oversight and monitoring

### Composition of the coordinating center and trial steering committee {5d}

#### Coordinating center

The trial is coordinated by the Chronic Viral Illness Service (CVIS) at the McGill University Health Centre (MUHC), Glen site, in Montreal, QC, under the supervision of the sponsor investigator, Dr. Jean-Pierre Routy. The coordinating center is responsible for day-to-day operations of the trial, including participant recruitment, data collection, protocol adherence, and communication with collaborating institutions.

#### Trial management team

A dedicated study team, including research coordinators, laboratory leads, and clinicians from both the MUHC and St. Joseph’s Health Care London, provides day-to-day support. This team is responsible for monitoring participant progress, managing data and sample logistics, coordinating study visits, and ensuring compliance with the protocol. The team meets as needed to address operational issues.

#### Trial steering committee (TSC)

The Gutsy study is overseen by a trial steering committee composed of key investigators, as well as representatives from CIHR/CTN. The TSC is composed of the PI, CTN project manager, CTN regulator affair specialist, CTN data manager, CTN statistician, study manager, clinical research coordinator, study nurse, and an external monitor. The TSC provides overall guidance and ensures the study remains aligned with its objectives, ethical standards, and regulatory compliance. The TSC is expected to meet quarterly throughout the trial, or more frequently if necessary.

#### Other committees

Data management and laboratory operations are handled by the MUHC Research Institute and collaborating research laboratories in London, ON. These teams are responsible for sample processing, secure data storage, and maintaining data quality standards.

### Composition of the data monitoring committee, its role and reporting structure {21a}

An independent Data Safety Monitoring Committee (DSMC) has been established to oversee participant safety and monitor trial data throughout the study. The DSMC is composed of independent experts in clinical trials, HIV research, and biostatistics, who are not involved in the conduct of the trial and have no competing interests with the sponsor investigator or funding bodies.

#### Role and responsibilities

The DSMC is responsible for periodic review of cumulative safety data and adverse events, advising on study continuation, modification, or early termination based on emerging safety concerns and ensuring that the rights and well-being of participants are protected.

#### Reporting structure

The DSMC will provide written recommendations to the sponsor investigator after each review meeting. These recommendations will also be communicated to the study team and the REB, as appropriate. The DSMC operates independently from the trial steering committee and sponsor.

#### Meetings and charter

The DSMC meets at regular intervals (e.g., every 6 months) or ad hoc if triggered by a safety event.

### Adverse event reporting and harms {22}

The study follows International Conference on Harmonisation Good Clinical Practice (ICH-GCP) guidelines for the collection, assessment, and reporting of adverse events (AEs).

#### Collection and assessment

Both solicited and spontaneously reported AEs are collected at each study visit. AEs are assessed for intensity (mild, moderate, or severe), causality (relationship to the study intervention), and expectedness. These assessments are recorded in the eCRF.

#### Serious adverse events (SAEs)

SAEs are defined per ICH guidelines and include events that are life-threatening, require hospitalization, result in disability, or death. All SAEs will be reported within 24 h to the medical monitor and sponsor investigator. Additional reporting to the REB will follow institutional and regulatory timelines.

#### Reporting and oversight

All AEs and SAEs will be monitored until resolution or stabilization. Pregnancy, if it occurs during the study, will be reported and followed to outcome. Unanticipated problems involving risk to participants will be reported promptly.

#### Data monitoring committee (DSMC)

The DSMC will review cumulative safety data at pre-specified intervals and make recommendations regarding trial continuation or modification.

### Frequency and plans for auditing trial conduct {23}

Periodic audits may be conducted to ensure compliance with the protocol, Good Clinical Practice (GCP) guidelines, and applicable Health Canada and institutional regulations. These audits are independent of the sponsor investigator and site investigators.

#### Auditing procedures and scope

Audits may be carried out by the CIHR Canadian HIV Trials Network (CTN), institutional quality assurance offices, or regulatory authorities. The audits may involve review of informed consent documentation, verification of source data and eCRF entries, assessment of adherence to inclusion/exclusion criteria, evaluation of adverse event reporting and safety documentation, inspection of investigational product storage, and accountability.

#### Frequency

Audits are anticipated at least once during the study, with additional visits conducted if required based on risk (e.g., protocol deviations, safety signals, or data irregularities).

#### Independence and corrective actions

Auditors will operate independently from the sponsor investigator and site staff. Findings will be documented and reported to the sponsor investigator and trial team. Any corrective actions required will be implemented and tracked in accordance with standard operating procedures.

### Plans for communicating important protocol amendments to relevant parties (e.g., trial participants, ethical committees) {25}

#### Prior ethics and sponsor approval required

No changes will be made to the protocol without prior approval from the Research Ethics Board (REB) and the sponsor investigator, except in cases where the change is necessary to eliminate an immediate hazard to participants.

#### Prompt reporting of changes and unanticipated problems

The investigator agrees to promptly report any changes in the research activity and all unanticipated problems involving risks to human participants or others to the applicable REBs.

#### Documentation of deviations

Any protocol deviations must be documented in the eCRF and reported accordingly.

#### Re-consent of participants

When amendments involve changes that affect participant safety or rights, the protocol implies that participants will be re-consented using an updated informed consent form, although this is standard practice even if not explicitly detailed in the text.

#### Investigators’ responsibilities

Investigators are responsible for adhering strictly to the protocol and for ensuring that any modifications are communicated to the entire study team and implemented only after appropriate approvals.

#### Trial registry and publication updates

adherence to ICH-GCP and Health Canada regulations, which require that trial registries (e.g., ClinicalTrials.gov) and, where applicable, journals and regulators be updated with significant amendments.

### Dissemination plans {31a}

#### Communication with participants

The study’s results will be published in open-access publication. All participants will be given access to the study findings. After study completion, participants will be informed of the overall trial results in study publications, press release, and study results presentations in local meetings.

#### Communication with the scientific and medical community

Results will be published in open-access peer-reviewed journals and presented at scientific conferences, irrespective of the outcome, including those related to HIV research (e.g., CTN semi-annual meetings).

#### Reporting to the public and regulatory databases

The trial is registered on ClinicalTrials.gov (Identifier: NCT06022406). Results are expected to be submitted to the registry upon study completion, as per Health Canada and ICH-GCP requirements.

#### Data sharing and publication restrictions

The protocol content is confidential and proprietary. Public disclosure must be authorized by the sponsor investigator (Dr. Jean-Pierre Routy). Investigators must adhere to publication policies agreed upon with the sponsor and funding agencies.

#### Data sharing arrangements

Since biological samples and data are stored for potential future testing, there is potential for future secondary analyses or data sharing. Any future data sharing would be governed by ethics approvals and data sharing agreements.

## Discussion

The Gutsy study (CTNPT 038) presents a novel and complex clinical trial evaluating the impact of FMT on immune activation in ART-treated PWH with low CD4/CD8 ratios. Several operational and practical considerations are important to highlight:

- Recruitment challenges: The study targets a very specific population of PWH (on stable ART for ≥3 years, CD4/CD8 ratio < 1), which may limit recruitment speed. Engagement with HIV clinics and community groups and presentations at HIV conferences are strategies proposed to address this challenge.
- FMT logistics: The use of encapsulated FMT introduces specific challenges: production, shipping under temperature control, storage at −80 °C, and direct observation of ingestion. Capsules are produced at a single site (St. Joseph’s Health Care, London, ON) and require coordination for timely delivery to the MUHC site.
- Blinding and randomization: The single-blind design, with randomization via sealed envelopes and varying capsule counts (30–40), may complicate implementation and require careful standard operating procedures to prevent unblinding.
- Optional colon biopsy sub-study: The invasive nature of colon biopsies may limit uptake for the sub-study. Operationally, this requires coordination with gastroenterology services, room scheduling, bowel preparation support, and post-procedure monitoring. Biopsy handling also demands careful processing, freezing or fixing, and coordination between multiple labs. However, this procedure may benefit participants with cancer screening procedure (44).
- Safety monitoring: Despite established safety in previous FMT studies, PWH represent a potentially immunocompromised population, so close monitoring is essential. A robust SAE reporting process is in place, and an independent DSMC oversees participant safety.
- Data management: The collection and storage of biological samples for both primary and exploratory endpoints require secure sample handling, proper biobanking, and detailed tracking systems. Data will be managed via eCRFs, and source documents will be retained per regulatory standards.
- Post-trial access and follow-up: The investigational product (FMT capsules) will not be provided after the trial. Participants will be informed of study findings but will not have continued access to treatment based on the investigational nature of the intervention.
- Regulatory and ethical oversight: All amendments and unexpected issues must be approved by the REB and sponsor investigator. Given the novel use of FMT in PWH, ongoing compliance with Health Canada and ICH-GCP is critical.

## Trial status

Current version of the protocol: Protocol V2.1 dated November 11, 2024, approved by Health Canada and the MUHC REB. Recruitment began in August 2024 and was completed in August 2025. Data collection is in progress.

## Supplementary Information

Additional file 1: Supplementary Table 1. Schedule of events.

## Data Availability

Access to the final trial dataset will be restricted to the core investigative team, including the sponsor investigator (JPR) and designated co-investigators (SI, MMS, SNP, BR), as well as approved members of the data analysis team at the McGill University Health Centre and St. Joseph’s Health Care, London. There are no contractual agreements that limit investigators’ access to the dataset. All data will be securely stored and managed in accordance with institutional policies and ethics board requirements. De-identified data may be made available to external researchers upon reasonable request, subject to REB approval and a signed data sharing agreement.

## Abbreviations

AE: Adverse event
AIDS: Acquired immune deficiency syndrome
ALT: Alanine transaminase
ART: Antiretroviral therapy
AST: Aspartate transaminase
CBC: Complete blood count
CCR: C-C chemokine receptor
CD: Cluster of differentiation
CHUM: Centre Hospitalier de l’Université de Montréal
CIHR: Canadian Institutes of Health Research
CMV: Cytomegalovirus
CRE: ESBL producing Enterobacteriaceae
CRP: C-reactive protein
CTA: Clinical trial application
CTN: CIHR Canadian HIV Trials Network
CVIS: Chronic Viral Illness Service
DNA: Deoxyribonucleic acid
eCRF: Electronic case report form
EIA: Enzyme immune assay
ELISA: Enzyme-linked immunosorbent assay
ESBL: Extended spectrum beta-lactamase
FDA: Food and Drug Administration
FMT: Fecal microbiota transplantation
HBV: Hepatitis B virus
HC: Health Canada
hCG: Human chorionic gonadotropin
HCV: Hepatitis C virus
HDL: High density lipoprotein
HIV: Human immunodeficiency virus
HLA-DR: Human leukocyte antigen-DR
hs-CRP: High-sensitivity C-reactive protein
IBD: Irritable bowel disease
ICJME: International Committee of Medical Journal Editors
I-FABP: Intestinal-fatty acid binding protein
IFN-γ: Interferon-gamma
IHMS: International Human Microbiota Standard
IL: Interleukin
IP-10: Interferon-gamma induced protein-10
LBP: Lipopolysaccharide binding protein
LDL: Low density lipoprotein
LNHPD: Licensed Natural Health Product Database
LPS: Lipopolysaccharide
mAbs: Monoclonal antibodies
MRSA: Methicillin-resistant *Staphylococcus aureus*
MUHC: McGill University Health Centre
NAFLD: Non-alcoholic fatty liver disease
NK: Natural killer (cell)
PBMC: Peripheral blood mononuclear cell
PD-1: Programmed cell death–1
PWH: People living with HIV
qPCR: Quantitative polymerase chain reaction
REB: Research Ethics Board
REG3α: Regenerative islet-derived protein 3 α
SAE: Serious adverse event
SARS-CoV-2: Severe acute respiratory syndrome coronavirus 2
SIV: Simian immunodeficiency virus
SJHC: St. Joseph’s Health Care London Hospital
sST2: Soluble suppression of tumorigenicity 2
Th17: T helper cell 17 (population of T cells)
TILDA: Tat/Rev Induced Limiting Dilution Assay
TNF-α: Tumor necrosis factor-alpha
