# Supplementary material for: Fecal microbiota transplantation to reduce immune activation in ART-treated people with HIV with low CD4/CD8 ratio: protocol for the single-blind, randomized, placebo-controlled Gutsy study (CIHR/CTN PT038)
Source: Trials. 2025 Dec 13;27:52. doi: 10.1186/s13063-025-09345-0 (PMC12821992; doi:10.1186/s13063-025-09345-0)
Supplement: Supplementary file 1 — Additional file 1: Supplementary Table 1. Schedule of events. [file 13063_2025_9345_MOESM1_ESM.docx]

**Supplementary Table 1: Schedule of Events.**

| **Visit Type** | **Screening** | **Study visits** | | | | | |
| --- | --- | --- | --- | --- | --- | --- | --- |
|  |  | **Baseline 1** | **Bowel cleanse** | **Baseline 2, Treatment 1** | **Treatment 2** | **Follow-up** | |
| **Visit Window**  **Procedures:** | **Visit 1**  **Day**  **-56 to -7 days** | **Visit 2**  **Day -14**  **(±7 days)** | **Day -1** | **Visit 3**  **Day 0** | **Visit 4**  **Day 22**  **(±7 days)** | **Visit 5**  **Week 6**  ***(±7 days)*** | **Visit 6**  **Week 12**  **(±7 days)** |
| Visit No. | 1 | 2 |  | 3 | 4 | 5 | 6 |
| Informed Consent | X |  |  |  |  |  |  |
| Eligibility Assessment | X | X |  | X |  |  |  |
| Concomitant Medication | X | X |  | X | X | X | X |
| Medical History | X |  |  |  |  |  |  |
| Complete Physical Exam and Vital Signs | X |  |  |  |  |  |  |
| Targeted Physical Examǂ and Vital Signs |  | X |  | X | X | X | X |
| Adverse Event Assessment |  |  |  | X | X | X | X |
| Serum Pregnancy Test | X | X |  | X | X | X | X |
| Hematology* | X | X^†^ |  | X | X | X | X |
| Serum Chemistry** | X | X^†^ |  | X | X | X | X |
| Serology*** | X |  |  | X |  |  |  |
| Serology - HIV-1 Viral Load*** | X | X^†^ |  | X | X | X | X |
| Randomization | X^††^ |  |  |  |  |  |  |
| Immune activation markers/cytokines (ELISA)**** |  | X |  | X | X | X | X |
| Monocyte and T cell activation markers^+^ |  | X |  | X | X | X | X |
| Markers of gut barrier integrity, immune activation and microbial translocation^++^ |  | X |  | X | X | X | X |
| Size of HIV reservoir in Latently Infected CD4 T cells^+++^ |  | X |  | X | X | X | X |
| Stool sample collection and microbiota composition^++++^ |  | X |  | X | X | X | X |
| Bristol Chart |  | X |  | X | X | X | X |
| Alcohol use (AUDIT-Full), Appendix 2 | X |  |  |  |  |  |  |
| Alcohol use (AUDIT-C), Appendix 3 |  | X |  | X | X | X | X |
| Bowel cleanse |  |  | X^†††^ |  |  |  |  |
| Study Product Dispensation (FMT or placebo) |  |  |  | X | X |  |  |
| Colon mucosal biopsies^#^ |  |  |  | X |  |  | X |

*CBC, CD4 and CD8 T cell counts.

**Alkaline phosphatase, ALT, Amylase, AST, Bilirubin (total), Creatine kinase, Creatinine, D-dimer, fasting blood glucose, HbA1c, high sensitivity C-reactive protein (hsCRP), Lipase, lipid profile (total cholesterol, high density lipoprotein (HDL), low density lipoprotein (LDL), Triglycerides), serum phosphate.

***Serology measurements include: Cytomegalovirus (CMV), Hepatitis B virus (HBV), Hepatitis C virus (HCV) and HIV viral load. Since HIV viral load are measured at each visit, it was put as a separate line item.

****Immune activation markers/cytokines include: soluble CD14, pro-inflammatory cytokines (IL-1β, IL-6, IL-8, TNF-α) and anti-inflammatory cytokine IL-10. Measured in plasma by ELISA.

+Monocyte and T cell activation markers include: HLA-DR and CD38. T cell exhaustion marker: PD-1. Measured by staining and flow cytometry.

++Markers of gut barrier integrity, microbial translocation and inflammation: lipopolysaccharide, soluble ST2, I-FABP (measured in plasma by ELISA).

+++PBMCs are isolated and then latent CD4 T cells are isolated by flow cytometry. HIV viral reservoir in the latent CD4 T cell population are measured by nested qPCR. More specific TILDA analysis will be performed on Baseline Week 0 and End-treatment Week 12 samples to assess the HIV viral reservoir (Exploratory analysis).

++++qPCR of A. muciniphila, 16S and 18S rDNA sequencing for other members of the microbiota.

# Optional sub-study procedure.

*^†^* Not required when the same tests have been performed at the screening visit within the past 14 days, with the exception of CBC, CD4, CD8 (and serum pregnancy test)

*^††^ upon validation of eligibility.*

*^†††^* Bowel cleanse the day before Visit 3 (Baseline 2).

*ǂ* If required.
